# Nano-Liposomal Carrier as Promising Dermal Delivery Platform for *Fumaria officinalis* L. Bioactives

**DOI:** 10.3390/pharmaceutics17060782

**Published:** 2025-06-14

**Authors:** Rabiea Ashowen Ahmoda, Milena Milošević, Aleksandar Marinković, Aleksandra A. Jovanović

**Affiliations:** 1 Faculty of Technology and Metallurgy, University of Belgrade, 11000 Belgrade, Serbia; 20214044@estudent.tmf.bg.ac.rs (R.A.A.); marinko@tmf.bg.ac.rs (A.M.); 2 Institute of Chemistry, Technology and Metallurgy—National Institute of the Republic of Serbia, University of Belgrade, 11000 Belgrade, Serbia; milena.milosevic@ihtm.bg.ac.rs; 3 Institute for the Application of Nuclear Energy INEP, University of Belgrade, 11080 Belgrade, Serbia

**Keywords:** bioactives, *Fumaria officinalis*, nanoliposomes, skin release kinetics, stability, sterols, rheology

## Abstract

**Background/Objectives:** This study investigates the physical, rheological, and antioxidant properties of nano-liposomal formulations encapsulating *Fumaria officinalis* L. (fumitory) extract, focusing on their stability and performance under ultraviolet (UV) exposure, as well as polyphenol release within simulated skin conditions in a Franz diffusion cell. **Methods:** Liposomal formulations, composed of phospholipids with or without β-sitosterol or ergosterol, were evaluated for their encapsulation efficiency, liposome size, size distribution, zeta potential, viscosity, surface tension, density, oxidative stability, antioxidant capacity, and polyphenol recovery. **Results:** Encapsulation efficiency was the highest in phospholipid liposomes (72.2%) and decreased with the incorporation of sterols: 66.7% for β-sitosterol and 62.9% for ergosterol liposomes. Encapsulation significantly increased viscosity and reduced surface tension compared to the plain liposomes, suggesting modified interfacial behavior. The inclusion of fumitory extract significantly increased the viscosity of liposomes (from ~2.5 to 6.09–6.78 mPa × s), consistent with the observed reduction in particle size and zeta potential. Antioxidant assays (thiobarbituric acid reactive substances—TBARS, 2,2′-azino-bis(3-ethylbenzothiazoline-6-sulfonic acid—ABTS, and 2,2-diphenyl-1-picrylhydrazyl—DPPH) confirmed enhanced lipid peroxidation inhibition and radical scavenging upon encapsulation, with ABTS activity reaching up to 95.05% in sterol-containing liposomes. Release studies showed that the free extract exhibited the fastest polyphenol diffusion (5.09 × 10^−9^ m^2^/s), while liposomes demonstrated slower/controlled release due to bilayer barriers. UV-irradiated liposomes released more polyphenols than untreated ones, particularly in the sterol-containing formulations, due to oxidative destabilization and pore formation. **Conclusions:** These findings highlight the potential of fumitory extract-loaded liposomes as stable, bioactive carriers with tunable polyphenol antioxidant release properties for dermal applications. Overall, liposomal formulations of fumitory extract exhibit significant potential for further development as a pharmaceutical, cosmetic, or dermo-cosmetic ingredient for use in the prevention and treatment of various skin disorders.

## 1. Introduction

The genus *Fumaria*, as a part of the Fumariaceae family, contains 46 species, including *Fumaria* species known as fumitory or earth smoke [1]. Due to a plethora of bioactive compounds, such as phenolics and alkaloids, *Fumaria officinalis* L., as a medicinal plant, was traditionally employed in Europe and Asia in the treatment of hepato-biliary, gastrointestinal, and skin disorders, as well as hypertension [2]. Its extracts possess antioxidant, anti-diabetic, antimicrobial, analgesic, anti-inflammatory, and cytoprotective effects [2,3]. Nevertheless, the utilization of polyphenol extracts and individual polyphenol compounds is limited due to their poor water solubility, resorption, and insufficient bioavailability [4]. Thus, numerous encapsulation techniques were established to overcome the mentioned problems and shortcomings [4,5]. A variety of conventional techniques have been well established and continue to be employed for the encapsulation of active ingredients. These include spray drying, spray-bed drying, fluid-bed coating, spray chilling, spray cooling, inclusion complexation with cyclodextrins, liposomal encapsulation, freeze drying (lyophilization), hot-melt extrusion, and melt injection [4]. Additionally, encapsulation processes using supercritical fluids, methods based on ionic interactions, technologies based on hydrophobic interactions, and chemical methods are also widely implemented for polyphenol components [5].

Lipid-based colloidal carriers, particularly liposomal vesicles, stand out as one of the most extensively utilized encapsulation systems in biomedicine. The mentioned lipid carriers containing spherical membranes, i.e., lipid bilayers, provide a specific combination of lipophilic and hydrophilic surroundings. Due to liposomes’ adaptability, bioavailability, ability to alter the size and number of layers, and biodegradability, as well as their wide range of potentially encapsulated components, liposomal vesicles can be transformed into remarkable constructs within the medical field for drug delivery systems [6,7,8]. Moreover, sterols like cholesterol, β-sitosterol, ergosterol, and stigmasterol can be added to liposomes to modify their bilayer membrane and its permeability, which can alter the final product’s characteristics and pharmacological behavior as well [9]. In particular, ergosterol improves the physical stability and membrane packing density of liposomal vesicles, which enhances their performance. In comparison to cholesterol, ergosterol has a higher degree of unsaturation; therefore, it can decrease membrane flexibility to a lesser extent [10]. Ergosterol-containing liposome bilayers are more fluid than those containing cholesterol, which, when combined with phospholipids, form a densely packed arrangement that significantly diminishes the efficacy of lipid ordering [11]. According to Yoda’s study [12], ergosterol improved the homogeneity of the liposome membrane with charged lipids. While liposomes containing ergosterol exhibited reduced reactions to charged lipids, membranes containing cholesterol are more susceptible to a charged state [13]. Nutraceuticals containing fungus sterols, such as ergosterol, can also support liver health, immunological function, and cardiovascular system well-being due to the reduction in cholesterol and triacylglyceride concentration in blood [14]. Moreover, the pharmacological effects of ergosterol related to the skin, such as antimicrobial, anti-inflammatory, antioxidant, and anticancer activities, have also been reported [14,15]. Namely, ergosterol shows potent skin barrier benefits by maintaining moisture balance and enhancing skin resilience without causing skin allergic reactions [14,16]. β-sitosterol is the most prevalent phytosterol that resembles structural similarities with cholesterol (possesses an additional ethylene group at the C-24 position) and shows anti-inflammatory, anti-diabetic, anti-cancer, immunomodulatory, and anti-hypercholesterolemic potential [17,18,19]. According to the literature data, higher proportions of β-sitosterol in phospholipid liposome bilayers lead to enhanced membrane rigidity, i.e., reduced permeability [18]. Lee et al.’s study [17] reported that β-sitosterol, as a phytosterol with positive physiological potential, exerted protective impacts on the stabilization of encapsulated bioactives in liposomal particles, supporting the broad implementation of phytosterols in the process of liposomal encapsulation. Regarding the skin’s beneficial effects and use in dermal products, β-sitosterol possesses anti-aging, immunomodulatory, antimicrobial, anti-inflammatory, wound healing, and antioxidant potential [16,20,21].

In our previous study [22], various *F. officinalis* extracts were developed and characterized in terms of chemical profile and biological potential. In the mentioned extracts, numerous polyphenol bioactives were identified, including caffeoylmalic and chlorogenic acids, quercetin, quercetin dihexoside, quercetin trihexoside, quercetin pentoside hexoside, methylquercetin pentoside hexoside, quercetin 3-*O*-rutinoside, methylquercetin deoxyhexosylhexoside, methylquercetin dihexoside, methylquercetin dihexoside, and kaempferol deoxyhexosylhexoside. Additionally, their antioxidant and anti-inflammatory capacity was demonstrated on the human keratinocyte cell line, with the absence of cytotoxicity [22]. To improve the stability and bioavailability of *F. officinalis* extract bioactives, as well as providing their controlled and prolonged release and activity on the skin, further experiments were performed. Therefore, the goals of the present study were (1) the development of phospholipid nano-liposomal carriers with *F. officinalis* extract in the absence and presence of a plant sterol (β-sitosterol) or a fungus sterol (ergosterol), (2) the physical characterization and stability monitoring of obtained carriers, (3) the determination of the antioxidant potential of obtained carriers, and (4) the investigation of phenolic release kinetics in simulated skin conditions.

## 2. Materials and Methods

### 2.1. Chemicals

The following phospholipids and sterols were used for liposome preparation: Phospholipon^®^ 90 G (fatty flakes, ≥94%, soybean unsaturated diacyl-phosphatidylcholine, Lipoid GmbH, Ludwigshafen, Germany) and β-sitosterol and ergosterol (Sigma-Aldrich, St. Louis, MO, USA). *F. officinalis* (aerial part) was purchased from the Institute for Medicinal Plant Research “Dr Josif Pančić” (Belgrade, Serbia). Ethanol (96%, REAHEM D.O.O. Srbobran, Serbia), 2,2′-azino-bis(3-ethylbenzothiazoline-6-sulphonic acid) or ABTS, 2,2-diphenyl-1-picrylhydrazyl or DPPH, thiobarbituric acid, perchloric acid, and phosphate-buffered saline (PBS) (Sigma-Aldrich, Darmstadt, Germany) were also used. For the liposome preparation, ultrapure water was used (Simplicity UV^®^ water purification system, Merck Millipore, Merck KGaA, Darmstadt, Germany).

### 2.2. Plant Extract Preparation

The extract was prepared using 0.5 g of intensively grinded plant material (particle size lower than 0.3 mm) and 70% *v*/*v* ethanol (15 mL) in a microwave extraction (microwave reactor, Monowave 300, Anton Paar, Graz, Austria) for 2 min at 100 °C, according to a previously published study [22]. The extract was filtered through filter paper.

### 2.3. Extract-Loaded Liposome Preparation

Liposomes with fumitory extract were formed in the proliposome procedure [8]. The extract, in a volume of 8 mL, was mixed with 2 g of pure phospholipids (Ph-liposomes) or the mixture of phospholipids and sterol (20 mol% of β-sitosterol or ergosterol, Ph-β-sitosterol and Ph-ergosterol liposomes, respectively) and heated to 60 °C for 30 min to evaporate ethanol and obtain a homogenous mixture. The addition of an aqueous medium (40 mL) in small portions was performed, and the whole volume was stirred at 1000 rpm for 2 h at ambient temperature. Unloaded liposomes (liposomal particles without extract) were prepared as a control. The procedure was the same as in the case of the extract-loaded liposomes, but instead of fumitory extract, the same amount of 70% *v*/*v* ethanol (8 mL) was added. The liposomes were kept in a refrigerator (4 °C) until future analyses.

### 2.4. Ultraviolet (UV) Irradiation of Liposomes

Developed liposomal systems (plain and extract-loaded liposomes), in a volume of 35 mL, were ultraviolet (UV)-irradiated in AC2-4G8, ESCo, Singapore, using an uncovered Petri dish for 20 min to test the impact of UV light exposure on their physical and rheological properties, stability, antioxidant effects, and release kinetics.

### 2.5. Encapsulation Efficiency

Before the determination of encapsulation efficiency, to separate the non-encapsulated fraction of *F. officinalis* extract from the loaded liposomes, their centrifugation was performed at 4 °C and 17,500 rpm for 45 min in a Thermo Scientific Sorval WX Ultra series ultracentrifuge (Thermo Fisher Scientific, Waltham, MA, USA). The amount of total polyphenols was determined spectrophotometrically (UV-1800, Shimadzu, Kyoto, Japan) in the supernatants using a modified Folin–Ciocalteu method [23]. The encapsulation efficiency was determined by the polyphenol concentration in the supernatant and calculated according to the following equation:

(1) encapsulation efficiency (%)=(TPCi−TPCsup)/TPCi×100

where TPC_i_ is the initial content of total polyphenols used for the preparation of liposomes, and TPC_sup_ is the content of total polyphenols determined in the supernatant. Encapsulation efficiency was determined on the 1st and 30th days (non-treated and UV-irradiated samples) and are expressed as a percentage.

### 2.6. Monitoring of the Plain and Extract-Loaded Liposome Storage Stability

The diameter of the liposome particles, as well as the index of polydispersity (PdI), and the zeta potential of the plain and *F. officinalis* extract-loaded liposomal systems (non-irradiated and UV-irradiated) were measured during 30 days of storage in a refrigerator (4 °C). Dynamic light scattering (photon correlation spectroscopy) was used for the measurements of the above-mentioned variables in a Zetasizer Nano Series device (Malvern Instruments, Malvern, UK). The measurements of all developed liposomes were repeated after 30 days in three repetitions at ambient temperature. Due to the requirements of this device, the liposome system was diluted 500 times, and 1 mL of diluted liposomes was used for the analyses.

### 2.7. An Examination of the Rheological Characteristics of the Plain and Extract-Loaded Liposomes

The plain and extract-loaded liposomal systems (non-treated and UV-irradiated) were transferred in a chamber with a spindle (Rotavisc lo-vi, IKA, Staufen, Germany) to determine their viscosity at ambient temperature during a rotation speed of 200 rpm. The measurements were performed in triplicate and repeated after 30 days of storage as well. The surface tension and density of the plain and extract-loaded liposomes were observed in the Force Tensiometer K20 (KRÜSS, Hamburg, Germany) device using a Wilhelmy plate and silicon crystal as the immersion body, respectively. The evaluations were carried out in triplicate and repeated after 30 days of storage.

### 2.8. Anti-ABTS and Anti-DPPH Radical Potential

The anti-radical potential of all developed extract-loaded liposomes and pure extract was examined by employing the ABTS and DPPH methods.

In the ABTS assay, 2 mL of the ABTS^•+^ solution was mixed with 20 µL of the liposomal suspension or pure extract (diluted to achieve the same concentration as in the liposome sample). The absorbance was read at 734 nm, after incubation in the dark (6 min), and calculated using the following equation:

(2) ABTS radical scavenging capacity (%)=(Ac−Ax)×100/Ac

where A_c_ is the absorbance of the control (ABTS^•+^ solution and water) and A_x_ is the absorbance of the ABTS^•+^ solution and liposomal sample or extract. All analyses were carried out in triplicate, and the anti-radical potential is expressed as the percentage of neutralization of free ABTS radicals (%).

In the DPPH method, an amount of 20 µL of the liposome sample or pure extract (diluted to achieve the same concentration as in the liposome sample) was mixed with the DPPH radical solution (2.8 mL). The absorbance was read at 517 nm, after incubation in the dark (20 min), and calculated as follows:

(3) DPPH radical scavenging capacity (%)=(Ac−Ax)×100/Ac

where A_c_ is the absorbance of the control (DPPH solution and water), and A_x_ is the absorbance of the DPPH solution and liposomal sample or extract. All analyses were carried out in triplicate, and the anti-radical potential is expressed as the percentage of neutralization of free DPPH radicals (%).

### 2.9. Thiobarbituric Acid-Reactive Substances Assay

In order to determine the peroxidation of three liposome types (Ph, Ph-β-sitosterol, and Ph-ergosterol) with encapsulated fumitory extract, the thiobarbituric acid-reactive substances (TBARS) assay was applied. Plain liposomes were used as a control. Both the control and liposomes with the extract were exposed to UV radiation for 12 h. Samples prepared from the same batch were stored in the dark and also used as a control. At certain time intervals during 12 h (at the 1st, 3rd, 5th, and 12th hours), 100 μL of liposome samples were taken and further used for a TBARS test [24]. The liposome aliquot was mixed with 1.5 mL of a 20% trichloroacetic acid solution and 1 mL of a stock solution (2% thiobarbituric acid and 20% perchloric acid, 1:1) in glass test tubes. The mixture was further heated in a water bath (Thermo Scientific Precision GP 10, Thermo Fisher Scientific, Waltham, MA, USA) at 100 °C. After 25 min, the test tubes were transferred to cold water in order to stop the reaction. An additional step of centrifugation (8 min at 3000 rpm) was conducted to remove the resulting precipitate. The characteristic pink color of the supernatant originated from a reaction between lipid hydroperoxide and thiobarbituric acid; thus, the absorbance was measured at 532 nm.

### 2.10. In Vitro Polyphenol Diffusion in Simulated Skin Conditions

In vitro polyphenol diffusion from the pure *F. officinalis* extract (diluted to achieve the same concentration as in the liposomal samples) and extract-loaded liposomes (non-treated and UV-irradiated Ph, Ph-β-sitosterol, and Ph-ergosterol liposomes) was investigated using a Franz diffusion cell (PermeGear, Hellertown, PA, USA). Phenolic release from the pure extract and extract-loaded liposomal vesicles in simulated skin conditions was monitored using an acetate cellulose membrane filter (pore size of 0.2 µm and diameter of 47 mm, Cytiva, Whatman, Maidstone, UK). The membrane filter separated the donor cell, with the sample (extract or various liposomes with extract), from the acceptor cell, with released and distributed polyphenols within the simulated medium (PBS, pH 7.4) at 35 °C using the procedure published by Abd et al. [25]. The extract or liposomes with extract in a volume of 2 mL were transferred to the filter membrane (donor compartment), while a PBS medium in the acceptor compartment was mixed at 800 rpm. The temperature was maintained at 35 °C, employing a water jacket and a peristaltic pump. The release kinetics were investigated for 24 h, and the sample in a volume of 350 µL was taken from the simulated medium in the receptor compartment at certain time intervals. The content of distributed phenolic compounds in the medium was measured using the direct spectrophotometric method, and the absorbance of the sample solution was read at 280 nm [26].

### 2.11. Statistical Data Processing

One-way analysis of variance and Duncan’s post hoc test (STATISTICA 7.0) were used for statistical data processing to ascertain whether there were statistically significant differences between the samples. All analyses were carried out in triplicate, and the data in the tables and graphs are shown as the mean ± standard deviation. Sample differences were deemed significant at *p* < 0.05. The data from the analysis of encapsulation efficiency, rheological characteristics, and antioxidant activity, as well as dynamic light scattering, were subjected to statistical data processing.

## 3. Results and Discussion

### 3.1. Encapsulation Efficiency of Fumaria officinalis Polyphenols in Developed Liposomes

The efficiency of the liposomal encapsulation process in entrapping the main bioactives from the *F. officinalis* extract formulation was measured. The data related to the determined encapsulation efficiency of Ph, Ph-β-sitosterol, and Ph-ergosterol liposomes (non-treated and UV-irradiated samples, at the 1st and 30th days of storage in a refrigerator) are shown in Table 1.

The encapsulation efficiency of liposomes for fumitory polyphenols ranged from 62.9% to 72.2% (Table 1). According to the literature data, the encapsulation efficiency of plant extracts into liposomal vesicles varied in a wide range [27,28,29]. For example, in comparison to data obtained in the present study, significantly higher values were found for the encapsulation of olive and chickweed extracts (88.40% and 84.25–92.09%, respectively) [28,29], which can be explained by a significant influence of liposomal membrane composition and the used herbal components on the encapsulation efficiency. On the other hand, the encapsulation efficiency of green tea extract in the optimal phosphatidylcholine liposomal formulation reached 53.58% [30], while the encapsulation efficiency of pennywort leaf extract amounted to 40.36–67.80% [27]. Regarding the impact of sterols (β-sitosterol and ergosterol) on encapsulation efficiency, one conclusion has arisen from the results presented in Table 1. Namely, this influence is unique, and the addition of sterols caused a significant drop in the encapsulation efficiency, from 72.2 ± 1.3% for Ph liposomes to 66.7 ± 1.1% for Ph-β-sitosterol liposomes and 62.9 ± 1.2% for Ph-ergosterol liposomes. The obtained drop can be explained by the effect of sterols on the structure of the liposomal bilayer. Hence, the incorporation of sterol within the liposome membrane can cause an increase in inter-lipid spacing [31,32] and consequently facilitate leakage of polyphenols from liposomal vesicles. Nevertheless, the encapsulation efficiency of the developed liposomes with fumitory extract did not significantly change after 30 days of storage in a refrigerator (Table 1).

UV lights can trigger the generation of reactive oxygen species (ROS), causing the creation of pores within the phospholipid membrane and, therefore, a significant leakage of encapsulated compounds, which has already been shown in previous studies [33,34]. However, in the case of fumitory extract-loaded liposomes, UV irradiation did not cause statistically significant changes in the encapsulation efficiency even after the 30-day storage (Table 1). The encapsulation efficiency measured in UV-irradiated samples was 64.1–71.9% on the 1st day and 63.0–70.8% on the 30th day. The reason for the absence of statistically significant differences between non-treated and UV-treated counterparts can lie in a short exposure of liposomes to UV light (20 min), as well as in the antioxidant (protective) effects of fumitory polyphenols encapsulated in liposomes, which can provide neutralization of free radicals generated by UV irradiation.

### 3.2. Physical Properties of Developed Liposomes

The physical properties of all developed liposomes (non-treated and UV-treated plain and fumitory extract-loaded lipid vesicles), including their vesicle diameter, PdI, and zeta potential, were measured immediately after the formulation and after 30 days of storage at 4 °C; the data are presented in Table 2.

The stability of the plain and *F. officinalis* extract-loaded liposomes (non-irradiated and UV-irradiated) was examined during 30 days of storage in a refrigerator via measurements of their diameter, size distribution, and zeta potential using photon correlation spectroscopy. Hence, photon correlation spectroscopy represents a technique for the rapid characterization of liposomal vesicles, which measures the time fluctuations of the light scattered by a liposomal population. Since liposomal particles move, i.e., Brownian motion, the light they scatter varies with time, and repeating the measurements for a high number of times provides average values [35]. Understanding and controlling the liposome size, shape, zeta potential, composition, as well as stability is crucial for optimizing the delivery of target bioactives and reaching desired therapeutic outcomes [7].

Liposomal particles, as phospholipid-based vesicles, show a spherical shape, and their diameters range from the nanoscale to the microscale (from 50 nm to 5 μm), containing single or multiple phospholipid bilayer shells, encasing a water chamber [36]. Size and zeta potential are often considered as the essential parameters that affect the fate of liposomes in vivo [7]. Hence, according to Edwards [37], liposomes with diameters ranging from 100 nm to 1 μm are termed large vesicles, while liposomes with a diameter > 1 μm are termed giant vesicles. Multilamellar liposomes possess two or more phospholipid bilayers and a size of 1–6 μm, and their size significantly affects their encapsulation efficiency and release kinetics [38]. The pharmacokinetic behavior and the half-life of liposomal vesicles are affected by their size as well [7]. As can be seen from Table 2, the diameter of all plain liposomes was significantly higher in comparison to their extract-loaded counterparts. The obtained data are consistent with the existing literature, which indicates that the diameters of liposomal vesicles are influenced by both the bilayer membrane composition and the characteristics and concentration of the encapsulated ingredients [39,40,41,42]. The observation that plain liposomes are significantly larger than their extract-loaded counterparts can be explained by several factors related to the interaction between the bioactives from fumitory extract and the lipid bilayer during liposome formation. Namely, changes in membrane packing and stability; surface charge, i.e., electrostatic repulsion; modulation of lipid phase behavior; as well as solvent and hydration effects can be responsible for the mentioned differences between unloaded and extract-loaded liposomal particles. According to the literature data, the behavior and motion of phospholipid groups are significantly affected in the presence of phenolic compounds due to their interactions between lipids and different molecules, like polyphenols, causing alterations in liposome polymorphism, size, or shape, as well as molecular exchange across the liposomal bilayer [43,44,45,46]. The literature shows that phenolic entrapment into liposomal spheres can decrease the amount of phospholipids participating in the liposome membrane’s creation, resulting in a decreased diameter of lipid vesicles. Namely, polyphenols can increase liposome membrane fluidity, while more fluid membranes favor liposomal particles with a smaller diameter [47,48]. Opposite results are shown in several studies, where liposomes loaded with plant extract possessed a higher size compared to unloaded liposomes [41,42,49,50], or where there was even the absence of differences between the size of empty and extract-loaded liposomes [51]. An explanation of these differences may lie in the various chemical characteristics of encapsulated compounds and, consequently, their position in the liposomal carrier. Namely, encapsulated ingredients, such as polyphenols, can be positioned in the aqueous core of liposomal vesicles (hydrophilic molecules), between hydrophobic phospholipid tails (lipophilic molecules) or at the bilayer surface (amphiphilic molecules), depending on their affinity, thus causing lesser or greater alterations in lipid particle diameter [49,52]. In addition, a selected technique for the encapsulation of extract into liposomal particles significantly affected the incorporation of active compounds, as well as liposome size. For example, lipophilic compounds can be entrapped in large quantities in the phospholipid bilayers at the beginning of the process of the liposomal vesicles’ self-assembly. On the other hand, hydrophilic compounds can be incorporated in lower amounts at the hydration stage, since one part is in the aqueous core of liposomes, whereas the other part remains outside the liposomal particles in the surrounding water [52,53]. Further, the size of plain Ph liposomes (sample without sterol) amounted to 420.6 ± 4.3 nm, while the addition of sterols caused a significant increase in liposome diameter (683.1 ± 12.9 nm for liposomes with β-sitosterol and 596.0 ± 15.6 nm for liposomes with ergosterol). The increase in vesicle diameter, observed upon sterol incorporation, is attributed to interactions between lipid acyl chains near the phospholipid headgroups, promoting inter-lipid spacing and resulting in membrane expansion [31,32]. UV irradiation did not change the liposome size, except for the plain and extract-loaded Ph liposomes, where the mentioned treatment could trigger modifications in liposome membrane conformation, i.e., an increase in liposome diameter [54,55].

In the plain liposomal population, the addition of sterols did not alter the values of PdI, and the mentioned variable varied in a narrow range, 0.205–0.259 (Table 2). The same trend was observed in the UV-irradiated plain liposomes, 0.206–0.256 (Table 2), indicating the presence of a uniform system. Hence, according to the literature data, PdI values < 0.2 are acceptable for polymer-based carriers, while PdI values < 0.3 are appropriate for liposomal carriers [56]. As PdI is an indicator of particle size distribution, it was anticipated that UV light irradiation would not impact the PdI, similar to its effect on the overall liposome population size. The addition of fumitory extract significantly increased the heterogeneity of the liposomal system. Specifically, the PdI values of extract-loaded liposomes were significantly higher compared to their plain counterparts, 0.289–0.302 (Table 2). The obtained results are expected, since Trucillo et al. [57] found that a higher amount of plant bioactives loading in liposomes resulted in larger dispersions, with PdIs increasing as the extract content within the liposomes rose. Moreover, in the case of Ph and Ph-ergosterol liposomes with fumitory extract, exposure to UV light significantly altered PdI values in comparison to non-treated samples, 0.398 and 0.373, respectively (Table 2). UV treatment notably enhanced the size heterogeneity of the fumitory extract-loaded Ph and Ph-ergosterol liposomes, which can be attributed to changes in membrane conformation. According to the literature data, the photochemical degradation of liposomes, induced by photon energy absorption, leads to significant alterations in the bilayer conformation. UV irradiation initiates changes in the physical properties of phospholipid bilayers by disrupting the order and packing of the phospholipids [54,55]. Furthermore, smaller liposomes (as liposomes with fumitory extract) exhibited higher PdI values compared to larger vesicles (as obtained plain liposomes) [9].

After the 30-day storage study, significant alterations in the liposome diameters were noticed only in the case of non-treated and UV-treated plain Ph liposomes (Table 2). Liposome viscosity represents a significant part of long-term storage and is a critical stability criterion [58]. Higher viscosity values of liposomes (as fumitory extract-loaded formulations compared to empty parallels, shown in Table 3) can provide a lower sedimentation rate, i.e., unchanged size distribution during storage, bearing greater system stability in the case of more viscous liposomal preparations [59]. The above-mentioned fact may explain the differences between the stability of unloaded and extract-loaded liposomes. Namely, liposomes with fumitory extract were more viscous (shown in Table 3), preventing the aggregation of lipid particles, i.e., changes in vesicle size. As can be seen in Table 2, the PdI values of all obtained liposomes remained unchanged up to the 30th day of storage.

Measurements of the charge of liposomes, as well as components selected for encapsulation, are essential for the prediction of membrane–membrane or membrane–component interactions and liposome susceptibility for aggregation. Specifically, charged liposomes, prepared by employing charged phospholipids (as in the case of all developed liposomes in the present study), are more stable in comparison to neutrally charged liposomes (such as the 1,2-dipalmitoyl-*sn*-glycero-3-phosphocholine and 1-palmitoyl-2-oleoyl-*sn*-glycero-3-phosphocholine samples), which show a tendency to fuse [43]. In the present study, all developed liposomal particles exhibited a negative zeta-potential. This could be attributed to the negative charge of polyphenols on the vesicle surface or to the increased exposure of negatively charged functional groups on the phospholipids [27]. The zeta potential of plain liposomes measured immediately after the preparation amounted to −17.6 mV (Ph liposomes), −20.6 mV (Ph-β-sitosterol liposomes), and −20.9 mV (Ph-ergosterol liposomes) (Table 2). Numerous studies have shown that the incorporation of sterols increases the spacing between phospholipid headgroups and enhances the hydrophobic stability of the liposomal membrane [27,60,61,62]. Consequently, the inclusion of sterols alters the ordering of the phospholipids and affects the thickness of the liposomal membrane, independent of the functional group nature in the phospholipids. These functional groups may also contribute to hydrogen bond formation with sterols and influence the zeta potential of the liposomes [62]. Hu et al. [63] reported that the addition of sterols within the liposomal membrane can enhance liposome stability by increasing their zeta potential, which boosts electrostatic repulsion between particles and thereby prevents the fusion and aggregation of liposome spheres. The described phenomenon was also noticed in the plain liposomes developed in the present study (Table 2). Liposomes with fumitory extract showed a significantly lower zeta potential: −5.99 mV (Ph liposomes), −6.11 mV (Ph-β-sitosterol liposomes), and −5.74 mV (Ph-ergosterol liposomes) (Table 2). This reduction is linked to interactions between the extract’s compounds and the liposome surface, which may modify the overall charge. According to the literature data, polyphenols encapsulated near the glycerol backbone of the phospholipid headgroup within the liposomal bilayer were found to enhance liposome colloidal stability. In contrast, compounds embedded deeper toward the bilayer’s core negatively impacted vesicle stability over time. Interestingly, molecules localized in the upper portion of the phospholipid acyl chains appeared to suppress liposomal aggregation and limit increases in vesicle size, potentially by promoting tighter packing among adjacent phospholipid molecules and increasing the exposure of phosphate headgroups at the bilayer surface [64]. UV treatment did not significantly affect the mentioned variable in both the plain and extract-loaded liposomes (Table 2). While UV exposure can significantly alter the zeta potential of liposomes, potentially even reversing it from negative to positive [65], no such change was observed with the liposomes prepared in this study. This can be explained by the fact that UV light typically does not induce a major reorganization of the phospholipid bilayer, disrupt membrane integrity, or cause leakage of encapsulated molecules, as confirmed by the measurements of encapsulation efficiency (Table 1).

### 3.3. Rheological Characteristics of Liposomes

The rheological properties of the plain and fumitory extract-loaded liposomes (non-treated and UV-irradiated formulations) were determined immediately after liposome formation and after 30 days of storage in a refrigerator via measurements of their viscosity, surface tension, and density. The results are presented in Table 3.

As can be seen in Table 3, the viscosity of all plain liposomes was significantly lower compared to their extract-loaded counterparts. The addition of fumitory extract in the liposomal system caused an increase in viscosity from ~2.5 mPa × s to 6.09–6.78 mPa × s. The incorporation of rosehip fruit extract in the liposomal formulation significantly raised the system viscosity compared to unloaded liposomes as well [66]. Shashidhar and Manohar [58] reported that a higher viscosity of liposome systems was an indication of smaller-sized liposomal particles. Namely, the fumitory extract-loaded liposomes possessed significantly lower diameters in comparison to unloaded parallels (Table 2), as well as higher viscosity values (Table 3). However, the implementation of sterols during liposomal preparation did not significantly affect the mentioned parameter. Hence, the viscosity of the liposomes obtained in the absence and the presence of sterols varied in a narrow range, which was expected, since liposomes show different flow behaviors, mostly depending on temperature [67], as well as the used surrounding medium, which was the same for all liposomal populations developed in the present study. UV irradiation did not lead to a notable alteration in the viscosity of the liposomal samples (plain and extract-loaded populations). Similar findings were reported by Demirbay et al. [68], who demonstrated that UV radiation had no significant effect on the viscosity of the solutions.

The viscosity values of all liposomal populations (plain and extract-loaded liposomes) altered after the 30-day storage, and measured values were significantly lower (1.57–1.87 mPa × s for plain samples and 4.98–5.17 mPa × s for liposomes with encapsulated fumitory extract, Table 3). The same trend was observed for UV-irradiated samples (1.76–2.00 mPa × s for plain liposomes and 4.84–5.60 mPa × s for liposomes with extract, Table 3). The observed reduction in viscosity values of the developed liposomes after 30 days of storage (as shown in Table 3) may contribute to the instability of the liposomal system. This could lead to changes in lipid vesicle size, including potential aggregation of spheres (an increase in liposome diameter) [59,69], which has already been shown in Section 3.2. for the non-treated and UV-irradiated empty Ph liposomes.

The surface tension, measured immediately after liposome formation, amounted to 20.3–23.8 mN/m for non-treated plain liposomes, 20.9–24.5 mN/m for UV-treated plain liposomes, 16.6–18.7 mN/m for non-treated liposomes with extract, and 17.0–18.2 mN/m for UV-irradiated liposomes with extract (Table 3). Surface tension was not affected by the incorporation of sterols into the liposomal system. Specifically, the surface tension was significantly lower in the presence of fumitory extract in the liposomal system. The observed decrease in surface tension may likely be attributed to the presence of surface-active compounds within the fumitory extract, such as alkaloids and flavonoids, which are commonly reported constituents of *Fumaria* species and are also found in *F. officinalis* extracts [22]. These phytochemicals can localize at the lipid–water interface and act similarly to natural surfactants, thereby reducing the interfacial tension between the aqueous and lipid phases during liposome formation. Azarbayjani et al. [70] showed that the surface tension of liposomal formulation is influenced by the phospholipid composition and is also affected by the characteristics and concentration of the entrapped compounds. These findings align with previous literature data, which suggest that polyphenols in the extracts may slightly reduce surface tension at the oil–water interface, thereby inhibiting lipid oxidation; however, they do not significantly enhance the system’s stability [71]. Conversely, Luo et al. [72] reported that flavonoids serve as effective emulsion stabilizers due to their ability to adsorb onto the surface. Moreover, the exposure to UV lights did not trigger changes in the surface tension of all developed liposomal formulations (Table 3), which is in agreement with the literature data [66].

The surface tension of all unloaded and fumitory extract-loaded liposomes significantly decreased after 30 days of storage in a refrigerator: 17.5–18.7 mN/m for non-treated and 16.9–18.6 mN/m for UV-irradiated empty liposomes and 13.9–15.0 mN/m for non-treated and 14.3–15.1 mN/m for UV-irradiated extract-loaded liposomes (Table 3). The changes in surface tension of the liposomal formulations after 30 days may be attributed to the formation of nanobubbles at the liquid surface. Specifically, the literature indicates that over time, the number of nanobubbles in the bulk liquid decreases, while the quantity of nanobubbles adsorbed at the liquid surface gradually increases. Consequently, the observed reduction in surface tension can be linked to the Janus-like structure of nanobubbles, which is capable of disrupting the hydrogen bonding network of water molecules at the liquid interface [73].

The density of empty liposomes and liposomal particles with extract was measured on the 1st and 30th days of storage at 4 °C (Table 3). The extract encapsulation in liposomes, as well as the incorporation of sterols, did not alter the density of the liposomal system, which varied in a narrow range, 0.997–1.003 g/cm^3^ (Table 3). The obtained data agree with the literature data [66,74,75]. The lack of a significant difference in the density values across the six liposomal populations was anticipated, as the density of liposomes and other liquid systems is influenced by the type and concentration of the phospholipids and solvents used in their formulation [76,77,78]. For the liposomes obtained in this study, an identical type of phospholipid (a commercial mixture) and the same medium for hydration (ultrapure water) were employed in all formulations. UV irradiation did not have a significant impact on the liposomal system density, as in the case of viscosity and surface tension. Furthermore, the density of all developed liposomes did not significantly change during the 30-day storage, 0.997–1.005 g/cm^3^ (Table 3).

### 3.4. Lipid Peroxidation in Formulated Liposomes

The TBARS assay was employed to evaluate the ability of the fumitory extract to inhibit or delay lipid peroxidation induced by UV irradiation. This assay is based on a reaction between thiobarbituric acid and malondialdehyde (MDA), a secondary byproduct generated during the degradation of lipid hydroperoxides, resulting in a chromogenic adduct measurable at 532 nm.

**Figure 1:**
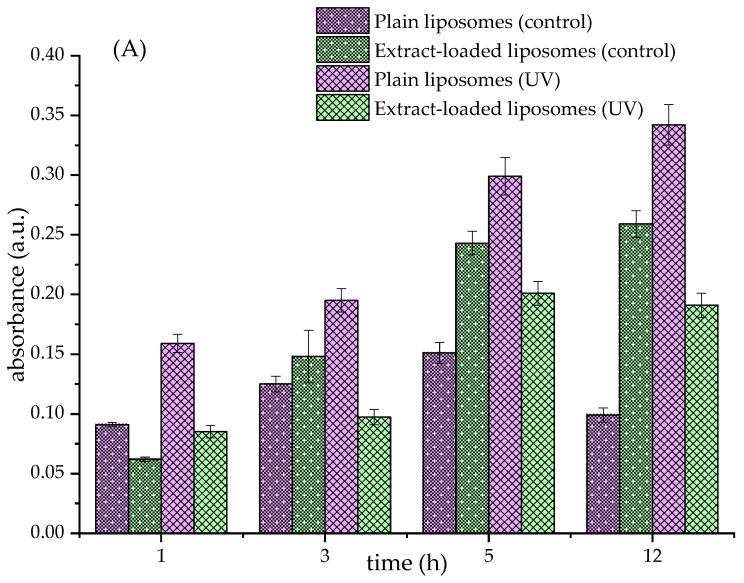

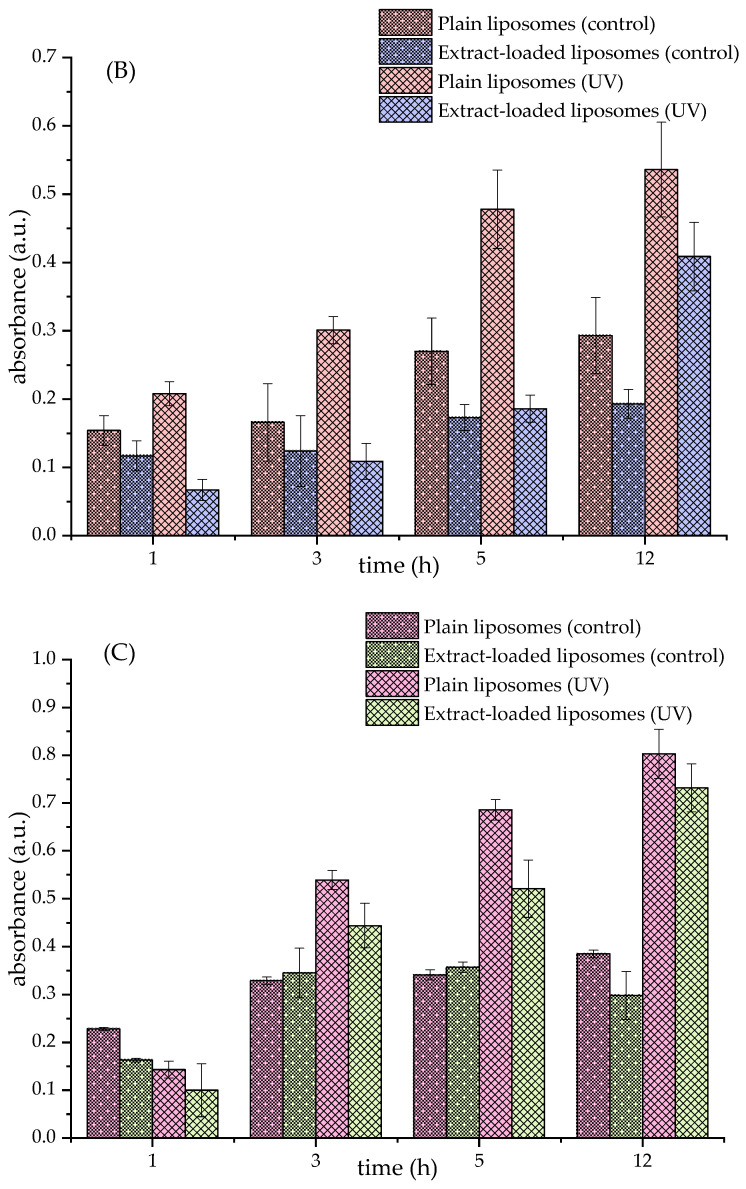
Effects of fumitory extract on liposomal oxidation (thiobarbituric acid-reactive substances assay, absorbance at 532 nm) under UV irradiation (UV) and stored in the dark (control). (**A**) Liposomes with pure phospholipids, (**B**) liposomes with phospholipids and 20 mol% of β-sitosterol, and (**C**) liposomes with phospholipids and 20 mol% of ergosterol.

Figure 1 presents the TBARS results for the various liposomal formulations, including Ph liposomes and those incorporating β-sitosterol or ergosterol. A marked increase in MDA levels was observed in UV-irradiated liposomes lacking the fumitory extract (plain liposomes) compared to formulations containing the extract or control liposomes stored under dark conditions. After 5 h of UV exposure, liposomes with encapsulated extract exhibited significantly reduced peroxidation levels, depending on the liposomal composition and demonstrating the antioxidant efficacy of the fumitory extract. These findings suggest that the extract offers protective benefits against oxidative degradation in liposomal systems composed of commercial lipid blends.

These observations are consistent with previous studies that have reported the antioxidative properties of polyphenolic compounds in mitigating lipid peroxidation [24,79]. However, the data presented in Figure 1B,C reveal that the inclusion of either β-sitosterol or ergosterol resulted in elevated lipid peroxidation levels under UV stress. This outcome aligns with our prior research, which established a concentration-dependent relationship between sterol content and lipid oxidation [9]. Specifically, while both sterols exhibit protective effects at lower concentrations, an increase beyond a certain threshold appears to reverse this trend, potentially due to the pro-oxidative behavior of sterols at higher levels. Their susceptibility to self-oxidation may contribute to the amplification of oxidative processes within the lipid bilayer.

### 3.5. Antioxidant Potential of Fumitory Extract-Loaded Liposomes

**Figure 2:**
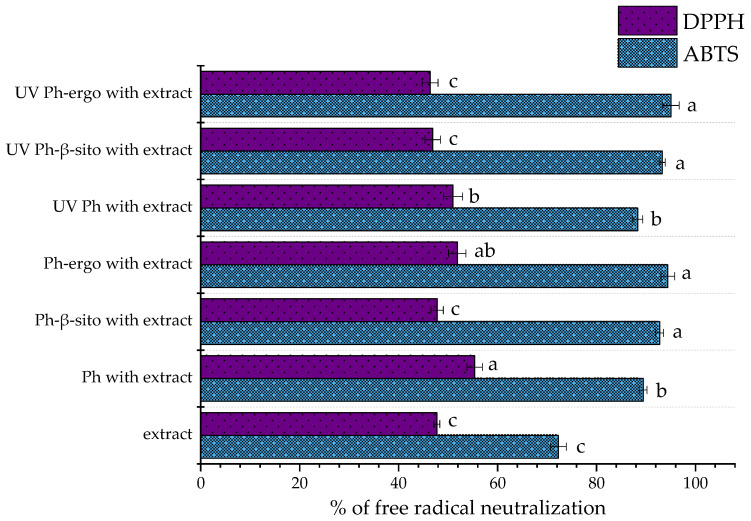
Radical scavenging activity of pure fumitory extract and fumitory extract-loaded liposomes (non-treated and UV-irradiated): DPPH and ABTS assays. Different letters (for each assay individually) indicate statistically significant differences as determined by Duncan’s post hoc test at *p* < 0.05 (n = 3; data are presented as the mean ± standard deviation). Liposomes with pure phospholipids, Ph; liposomes with phospholipids and 20 mol% of β-sitosterol, Ph-β-sito; liposomes with phospholipids and 20 mol% of ergosterol, Ph-ergo.

The radical scavenging activity of the pure fumitory extract and its corresponding liposomal formulations (non-treated and UV-irradiated samples) is illustrated in Figure 2. The antioxidant potential, as assessed through both DPPH and ABTS assays, is expressed as the percentage of neutralized free radicals, reflecting the extract’s and formulations’ efficiency in mitigating oxidative stress.

Regarding the ABTS assay, the antioxidant activity of the free fumitory extract (diluted to match the concentration used in the liposomal formulations) was determined to be 72.30 ± 1.60% (Figure 2). In contrast, Ph liposomes loaded with the fumitory extract demonstrated a significantly greater scavenging capacity (89.43 ± 0.79% for non-treated and 88.33 ± 1.00%). Ph liposomes with sterols loaded with the fumitory extract also possessed significantly higher free radical neutralization activity. Hence, Ph-β-sitosterol liposomes neutralized 92.77 ± 0.78% (non-treated sample) and 93.28 ± 0.61% (UV-irradiated sample), while Ph-ergosterol liposomes scavenged 94.43 ± 1.39% (non-treated sample) and 95.05 ± 1.67% (UV-irradiated sample) of free ABTS radicals.

These results are consistent with other reports highlighting enhanced antioxidant activity upon encapsulation. For instance, Noudoost et al. [80] observed increased antioxidant potential in liposome-encapsulated green tea extract compared to its free counterpart, while Jahanfar et al. [50] found greater inhibitory effects in rosemary-loaded glycerosomes than in non-encapsulated extract. Zokti et al. [81] reported that encapsulation efficiency can be influenced by the nature of wall materials, affecting antioxidant activity as well. The modification of the extract’s antioxidant capacity after encapsulation within liposome carriers was expected. Namely, new physicochemical characteristics, as well as the changed biological potential of the complex created between the liposome membrane and bioactives, occur and depend on the composition, size, and surface charge of developed liposomal populations [82]. Spigno et al.’s study [83] showed that encapsulation of a phenolic grape marc extract in nanosystems enhanced phenolic efficiency against lipid oxidation by increasing their dispersibility in the environment and preserving antioxidant activity. Additionally, Cortie and Else [84] proposed that non-peroxidizable phospholipids could exert intrinsic antioxidant-like effects within lipid membranes, potentially complementing the activity of encapsulated antioxidant agents. Prior studies have also shown that liposomes made from phospholipid blends containing antioxidants exhibit notable radical scavenging properties even in the absence of added active compounds [8], which may explain the higher antioxidant activity of the fumitory-loaded liposomes compared to the free extract. The significantly higher antioxidant potential in the ABTS assay of liposomes containing β-sitosterol or ergosterol in comparison to Ph liposomal parallels may be explained by previously mentioned antioxidant effects of these sterols [14,15,20].

The DPPH assay revealed that the pure fumitory extract (diluted to match the concentration used in the liposomal formulations) exhibited a radical scavenging activity of 47.73 ± 0.60% (Figure 2). Higher values were observed in both untreated and UV-exposed Ph liposomes (55.37 ± 1.54% and 51.00 ± 1.95%, respectively) and untreated Ph-ergosterol liposomes (51.87 ± 1.76%). Comparable values with data related to pure extract were observed in both non-treated and UV-exposed Ph-β-sitosterol liposomes and UV-irradiated Ph-ergosterol liposomes (47.79 ± 1.25%, 46.89 ± 1.57%, and 46.37 ± 1.61%, respectively), indicating that the encapsulation process effectively preserved the extract’s antioxidant functionality. These findings suggest that the formulated liposomal systems are capable of maintaining the DPPH scavenging capacity of the fumitory extract post-encapsulation. Likewise, UV exposure may induce ROS-mediated damage to the lipid bilayer [33], further compromising the antioxidant performance of the liposomal formulations, which could be the case with Ph and Ph-ergosterol liposomes, whose non-treated samples showed better antioxidant potential (Figure 2). The differences between the data obtained in both employed anti-radical tests were expected due to differences related to the characteristics and reactivity of free radicals and conditions during the experiment (time, pH value, etc.).

### 3.6. Polyphenol Release Kinetics from Fumitory Extract and Extract-Loaded Liposomes in Simulated Skin Conditions

**Figure 3:**
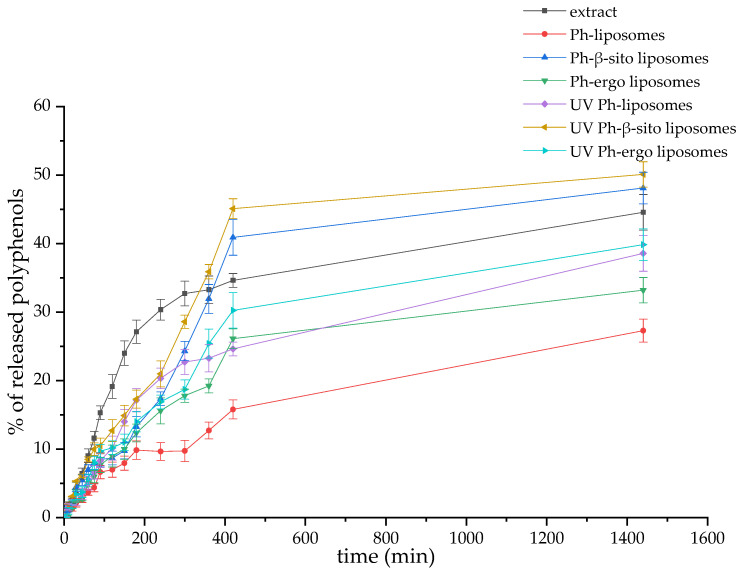
Polyphenol release kinetics from fumitory extract and fumitory extract-loaded liposomes (non-treated and UV-irradiated), monitored at 35 °C in a Franz diffusion cell using simulated skin conditions (phosphate-buffered saline, pH 7.4) for 24 h. Liposomes with pure phospholipids, Ph-liposomes; liposomes with phospholipids and 20 mol% of β-sitosterol, Ph-β-sitosterol liposomes; liposomes with phospholipids and 20 mol% of ergosterol, Ph-ergo liposomes.

The release behavior of polyphenolic compounds from both the free fumitory extract and its liposome-encapsulated forms (including untreated and UV-exposed formulations) was investigated under simulated skin conditions, specifically, a PBS medium (pH 7.4) at 35 °C. The corresponding release profiles are illustrated in Figure 3. To better understand the release dynamics, the diffusion coefficients and diffusion resistances associated with polyphenol transport from the liposomal systems were calculated. These calculations, which provide insights into the release kinetics within the skin-simulated medium, are detailed in the Supplementary Materials. The computed values for the diffusion parameters are summarized in Table 4.

As illustrated in Figure 3, the release of polyphenolics in simulated skin conditions was notably higher and more rapid from the free fumitory extract compared to both non-treated and UV-exposed liposomes. After a 24 h period, the cumulative release reached 44.57 ± 2.60% for the pure extract, 27.30 ± 1.69% for Ph-liposomes, 48.11 ± 2.33% for Ph-β-sitosterol liposomes, and 33.20 ± 1.85% for Ph-ergosterol liposomes (Figure 3). Furthermore, UV-irradiated liposomes released significantly higher amounts of polyphenols in comparison to their non-treated counterparts, at 38.57 ± 2.2%, 50.10 ± 1.95%, and 39.85 ± 2.2% (Figure 3).

The calculated diffusion coefficients reflect these trends. Non-treated liposomes exhibited similar diffusion rates, measured at 3.48 × 10^−9^ m^2^/s (for Ph-liposomes), 4.02 × 10^−9^ m^2^/s (Ph-β-sitosterol liposomes), and 4.30 × 10^−9^ m^2^/s (Ph-ergosterol liposomes) (Table 4). In comparison, the free extract displayed a markedly higher diffusion coefficient of 5.09 × 10^−9^ m^2^/s (Table 4). Correspondingly, diffusion resistance values followed the inverse pattern: the free extract exhibited lower resistance (8.01 × 10^5^ s/m), whereas the non-treated liposomal samples showed higher resistances (1.17 × 10^6^ s/m, 9.48 × 10^5^, and 1.43 × 10^6^ s/m), as detailed in Table 4.

UV-irradiated liposomes showed a higher diffusion rate than non-treated parallels, which amounted to 5.42 × 10^−9^ m^2^/s (Ph-liposomes), 1.10 × 10^−8^ m^2^/s (Ph-β-sitosterol liposomes), and 9.64 × 10^−9^ m^2^/s (Ph-ergosterol liposomes) (Table 4). Consequently, the diffusion resistance values followed the inverse pattern: 7.51 × 10^5^ s/m for Ph-liposomes, 4.23 × 10^5^ for Ph-β-sitosterol liposomes, and 1.43 × 10^6^ s/m for Ph-ergosterol liposomes, as presented in Table 4.

Due to the sensitivity of polyphenols to oxygen and surrounding environmental conditions, higher release levels were obtained from non-treated and UV-irradiated Ph-β-sitosterol formulations in comparison to pure fumitory extract. Additionally, the presence of various sterols in liposomal preparations can alter the rigidity and permeability of the liposome bilayer [9,85]. Liposomes containing β-sitosterol or ergosterol showed a higher release of fumitory polyphenols compared to Ph-liposomes. Sterols can impact the mechanical characteristics of the liposomal bilayer and, consequently, the delivery of bioactives from the liposomal particles because of the changes in the acyl chain order; however, this effect is not universal and depends on the bilayer composition and amount of sterols [86,87]. Liposomes of various sizes are also reported to have differences in the release kinetics of encapsulated bioactives. Owing to their higher hydrodynamic diameter, multilamellar vesicles accommodate a greater entrapped volume than smaller unilamellar particles, and thus, small liposomes (~100 nm) exhibit a faster mass transfer in comparison to larger vesicles [7,52]. However, in the case of the fumitory extract-loaded liposomes, none of the samples belongs to the class of unilamellar liposomes; therefore, the variations in release rate are not associated with the number of phospholipid bilayers, i.e., barriers that the target compounds have to overcome altogether to be released. The higher polyphenol release from the UV-irradiated liposomes with β-sitosterol or ergosterol compared to Ph-liposomes can be explained by the previously mentioned susceptibility of sterols to oxidation, which can contribute to the amplification of oxidative reactions within the liposomal membranes, as well as the formation of pores. Namely, the significantly improved diffusivity and reduced resistance observed in UV-treated liposomes compared to untreated ones may be attributed to UV-induced membrane perturbations, such as pore formation within the lipid bilayers [34]. This explanation aligns with previous studies reporting the enhanced release of encapsulated bioactives from liposomes following UV-triggered destabilization of the vesicle structure [88,89,90]. The viscosity of liposomes has a significant function in efficient drug release. Namely, it is known that liposomal systems with high viscosity values show slower encapsulated ingredient distribution and, consequently, a lower clearance rate after administration [58]. However, there were no statistically significant differences between the viscosity of all developed liposomes with fumitory extract, and all samples showed low viscosity values. Specifically, when viscosity possesses lower values, molecules diffuse more effectively, allowing for more enhanced release of the compounds in the surrounding environment [91]. Additionally, with the aim of investigating potential structural changes in the developed liposomes, as well as their thermal properties under UV irradiation, future research should encompass Fourier transform infrared (FT-IR) and Raman spectroscopy and differential scanning calorimetry, respectively [92].

## 4. Conclusions

This study investigated the encapsulation efficiency, size, size distribution, zeta potential, stability, and antioxidant potential of liposomes containing fumitory extract, with or without sterol incorporation, under refrigerated storage and UV irradiation conditions. The novel aspect of this work lies in the detailed investigation of how sterols such as β-sitosterol and ergosterol influence liposomal characteristics, encapsulation efficiency, and antioxidant performance, an area that has not been extensively explored for fumitory extract delivery. Encapsulation efficiency was the highest in Ph-liposomes (~72%) and decreased with the incorporation of β-sitosterol and ergosterol (~66% and ~62%, respectively), due to sterol-induced bilayer perturbation and increased inter-lipid spacing, facilitating polyphenol leakage. The liposome diameter was significantly reduced in the extract-loaded samples in comparison to plain parallels (from 420–683 nm to 270–345 nm) and enhanced with the incorporation of β-sitosterol or ergosterol (from 420 nm to 596–683 nm and from 270 nm to 311–345 nm). The PdI values show that plain liposomes were well-monodispersed (0.205–0.259), but their uniformity decreased when loaded with extract (0.289–0.302) and exposed to UV light (0.319–0.398). The zeta potential analysis indicated that all formulations had a negative surface charge, which became less negative after encapsulating the extract (~−6 mV). The inclusion of sterols enhanced the zeta potential only in the plain liposomes (~−20 mV), contributing to colloidal stability, while the zeta potential of the extract-loaded liposomes was insufficient. The present study confirms that liposomal encapsulation of fumitory extract significantly enhances the antioxidant efficacy of the extract, while also allowing for controlled polyphenol release under simulated skin conditions. Encapsulation increased radical scavenging activity, as confirmed by ABTS and DPPH assays, and markedly reduced lipid peroxidation, as shown in TBARS measurements. Furthermore, polyphenol release from the liposomes was slower and more sustained compared to the free extract, with diffusion coefficients and resistance values supporting improved barrier properties of the liposomal bilayer. UV irradiation notably increased release and diffusion rates, particularly in sterol-containing liposomes, due to potential membrane destabilization and pore formation. Among the tested formulations, β-sitosterol- and ergosterol-containing liposomes exhibited higher anti-ABTS radical activity and polyphenol diffusivity, highlighting the sterol-dependent modulation of membrane dynamics. All developed liposomes demonstrated low viscosity (2–6 mPa × s), supporting their potential for dermal delivery without compromising release efficiency. Importantly, this formulation strategy provides protection against oxidative degradation and allows for controlled, sustained delivery under conditions simulating skin exposure, such as UV irradiation. Our findings demonstrate that liposomal encapsulation significantly improves the stability, antioxidant efficacy, and controlled release of polyphenols from fumitory extract. Notably, extract-loaded liposomes exhibited a reduced particle size and sustained release profiles, which are crucial for enhanced bioavailability and therapeutic performance. The incorporation of sterols modulated membrane properties, affecting the liposome size, surface charge, and polyphenol release kinetics, with β-sitosterol- and ergosterol-containing liposomes showing superior radical scavenging activity and controlled diffusion. These findings underscore the value of liposomal carriers for delivering fumitory extract, offering protection against oxidative degradation and enabling controlled release of bioactives, making them promising candidates for topical antioxidant therapies and supporting their application in pharmaceutical or dermo-cosmetic formulations. Overall, this research introduces a promising and innovative liposomal delivery platform for fumitory extract, leveraging natural sterol–lipid interactions to optimize carrier performance and bioactive efficacy. These advantages position the developed liposomes as effective and safe candidates for future topical antioxidant formulations, contributing valuable insights into plant extract encapsulation in nanocarriers. Future experiments will include the monitoring of storage stability at ambient temperature, thermal properties of the developed liposomes, and chemical characterization by using Fourier transform infrared and Raman spectroscopy and high-performance liquid chromatography. Furthermore, a nanoparticle tracking analyzer and transmission electron microscopy will be employed for additional investigations.

## Figures and Tables

**Table 1:** Encapsulation efficiency of Ph, Ph-β-sitosterol, and Ph-ergosterol liposomes with *Fumaria officinalis* extract before and after UV irradiation, measured on the 1st and 30th days of storage at 4 °C.

Day	Liposomes		Encapsulation Efficiency (%)
1st	Non-treated	Ph	72.2 ± 1.3 ^a^*
		Ph-β-sitosterol	66.7 ± 1.1 ^b^
		Ph-ergosterol	62.9 ± 1.2 ^c^
	UV-irradiated	Ph	71.9 ± 0.5 ^a^
		Ph-β-sitosterol	68.7 ± 1.5 ^b^
		Ph-ergosterol	64.1 ± 1.0 ^c^
30th	Non-treated	Ph	73.0 ± 2.1 ^a^
		Ph-β-sitosterol	65.5 ± 1.0 ^b^
		Ph-ergosterol	61.4 ± 0.9 ^c^
	UV-irradiated	Ph	70.8 ± 1.9 ^a^
		Ph-β-sitosterol	66.4 ± 2.0 ^b^
		Ph-ergosterol	63.0 ± 1.7 ^bc^

* The same letter refers to the absence of statistically significant differences regarding the results of the statistical analysis in a one-way analysis of variance and Duncan’s post hoc test at *p* < 0.05 (n = 3). Liposomes with pure phospholipids, Ph; liposomes with phospholipids and 20 mol% of β-sitosterol or ergosterol, Ph-β-sitosterol and Ph-ergosterol, respectively.

**Table 2:** Diameter, polydispersity index (PdI), and zeta potential of Ph, Ph-β-sitosterol, and Ph-ergosterol liposomes with and without *Fumaria officinalis* extract before and after UV irradiation, measured on the 1st and 30th days of storage at 4 °C using dynamic light scattering.

Day	Liposomes		Diameter (nm)	PdI	Zeta Potential (mV)
1st	Non-treated	Plain Ph	420.6 ± 4.3 ^g^*	0.259 ± 0.013 ^c^	−17.6 ± 0.3 ^b^
		Plain Ph-β-sitosterol	683.1 ± 12.9 ^a^	0.205 ± 0.026 ^d^	−20.6 ± 0.6 ^a^
		Plain Ph-ergosterol	596.0 ± 15.6 ^cd^	0.232 ± 0.017 ^cd^	−20.9 ± 0.5 ^a^
		Ph with extract	270.6 ± 5.5 ^k^	0.302 ± 0.026 ^b^	−5.99 ± 0.62 ^d^
		Ph-β-sitosterol with extract	311.7 ± 13.5 ^i^	0.289 ± 0.020 ^bc^	−6.11 ± 1.03 ^d^
		Ph-ergosterol with extract	345.8 ± 9.5 ^h^	0.295 ± 0.048 ^bc^	−5.74 ± 0.89 ^d^
	UV-irradated	Plain Ph	442.8 ± 5.5 ^f^	0.253 ± 0.030 ^cd^	−17.3 ± 0.4 ^b^
		Plain Ph-β-sitosterol	667.2 ± 13.0 ^a^	0.206 ± 0.020 ^d^	−20.9 ± 0.8 ^a^
		Plain Ph-ergosterol	586.7 ± 16.0 ^cd^	0.256 ± 0.021 ^cd^	−19.1 ± 1.7 ^ab^
		Ph with extract	291.9 ± 2.6 ^j^	0.398 ± 0.019 ^a^	−5.31 ± 0.29 ^d^
		Ph-β-sitosterol with extract	315.1 ± 7.1 ^i^	0.319 ± 0.025 ^b^	−5.69 ± 0.99 ^d^
		Ph-ergosterol with extract	354.8 ± 10.2 ^h^	0.373 ± 0.018 ^a^	−6.03 ± 0.54 ^d^
30th	Non-treated	Plain Ph	558.0 ± 11.2 ^e^	0.273 ± 0.049 ^bc^	−17.1 ± 0.2 ^b^
		Plain Ph-β-sitosterol	663.3 ± 9.5 ^a^	0.223 ± 0.052 ^cd^	−18.0 ± 0.4 ^b^
		Plain Ph-ergosterol	616.2 ± 23.3 ^bc^	0.242 ± 0.059 ^cd^	−16.1 ± 1.2 ^bc^
		Ph with extract	279.8 ± 4.9 ^k^	0.375 ± 0.050 ^ab^	−5.65 ± 0.56 ^d^
		Ph-β-sitosterol with extract	326.4 ± 14.3 ^hi^	0.287 ± 0.019 ^bc^	−5.48 ± 1.12 ^d^
		Ph-ergosterol with extract	350.1 ± 11.1 ^h^	0.351 ± 0.025 ^ab^	−6.14 ± 1.04 ^d^
	UV-irradated	Plain Ph	581.6 ± 3.4 ^d^	0.269 ± 0.038 ^bc^	−16.9 ± 0.9 ^bc^
		Plain Ph-β-sitosterol	658.7 ± 31.9 ^ab^	0.201 ± 0.086 ^d^	−16.3 ± 0.4 ^c^
		Plain Ph-ergosterol	622.9 ± 25.4 ^bc^	0.295 ± 0.023 ^bc^	−15.4 ± 0.7 ^c^
		Ph with extract	294.3 ± 4.2 ^j^	0.389 ± 0.008 ^a^	−5.48 ± 0.06 ^d^
		Ph-β-sitosterol with extract	328.8 ± 17.5 ^hi^	0.272 ± 0.047 ^bc^	−5.91 ± 0.84 ^d^
		Ph-ergosterol with extract	346.9 ± 10.1 ^h^	0.385 ± 0.021 ^a^	−5.63 ± 1.09 ^d^

* The same letter in each column refers to the absence of statistically significant differences regarding the results of the statistical analysis in a one-way analysis of variance and Duncan’s post hoc test at *p* < 0.05 (n = 3). Liposomes with pure phospholipids, Ph; liposomes with phospholipids and 20 mol% of β-sitosterol or ergosterol, Ph-β-sitosterol and Ph-ergosterol, respectively.

**Table 3:** Rheological properties of plain and *Fumaria officinalis* extract-loaded liposomes (non-treated and UV-irradiated), examined on the 1st and 30th days of storage at 4 °C.

Day		Liposomes	Viscosity (mPa × s)	Surface tension (mN/m)	Density (g/cm^3^)
1st	Non-treated	Plain Ph	2.58 ± 0.11 ^c^*	23.8 ± 1.0 ^a^	0.998 ± 0.002 ^a^
		Plain Ph-β-sitosterol	2.43 ± 0.29 ^c^	22.6 ± 1.3 ^ab^	0.999 ± 0.000 ^a^
		Plain Ph-ergosterol	2.57 ± 0.17 ^c^	20.3 ± 1.4 ^bc^	1.001 ± 0.002 ^a^
		Ph with extract	6.09 ± 0.33 ^a^	17.6 ± 0.5 ^d^	1.000 ± 0.001 ^a^
		Ph-β-sitosterol with extract	6.78 ± 0.45 ^a^	16.6 ± 0.9 ^d^	1.002 ± 0.005 ^a^
		Ph-ergosterol with extract	6.49 ± 0.22 ^a^	18.7 ± 1.3 ^cd^	0.998 ± 0.003 ^a^
	UV-irradiated	Plain Ph	3.04 ± 0.51 ^c^	24.5 ± 0.5 ^a^	1.001 ± 0.001 ^a^
		Plain Ph-β-sitosterol	3.10 ± 0.38 ^c^	21.7 ± 0.9 ^ab^	1.000 ± 0.001 ^a^
		Plain Ph-ergosterol	2.92 ± 0.27 ^c^	20.9 ± 0.7 ^b^	1.003 ± 0.005 ^a^
		Ph with extract	6.31 ± 0.50 ^a^	18.2 ± 0.8 ^cd^	0.997 ± 0.004 ^a^
		Ph-β-sitosterol with extract	6.92 ± 0.43 ^a^	17.0 ± 1.1 ^d^	1.000 ± 0.001 ^a^
		Ph-ergosterol with extract	6.81 ± 0.38 ^a^	17.9 ± 0.5 ^d^	0.999 ± 0.004 ^a^
30th	Non-treated	Plain Ph	1.87 ± 0.22 ^d^	17.5 ± 1.1 ^d^	1.004 ± 0.002 ^a^
		Plain Ph-β-sitosterol	1.69 ± 0.15 ^d^	18.7 ± 1.3 ^cd^	1.005 ± 0.004 ^a^
		Plain Ph-ergosterol	1.57 ± 0.29 ^d^	17.8 ± 1.0 ^d^	1.002 ± 0.005 ^a^
		Ph with extract	5.17 ± 0.40 ^b^	14.2 ± 0.9 ^f^	0.999 ± 0.003 ^a^
		Ph-β-sitosterol with extract	4.98 ± 0.83 ^b^	13.9 ± 1.0 ^f^	1.000 ± 0.001 ^a^
		Ph-ergosterol with extract	5.06 ± 0.64 ^b^	15.0 ± 0.9 ^ef^	0.999 ± 0.004 ^a^
	UV-irradiated	Plain Ph	2.00 ± 0.12 ^d^	18.6 ± 1.2 ^cd^	1.000 ± 0.001 ^a^
		Plain Ph-β-sitosterol	1.76 ± 0.31 ^d^	18.0 ± 0.7 ^cd^	1.000 ± 0.003 ^a^
		Plain Ph-ergosterol	1.97 ± 0.13 ^d^	16.9 ± 1.2 ^de^	0.997 ± 0.005 ^a^
		Ph with extract	5.60 ± 0.15 ^b^	15.1 ± 1.0 ^ef^	1.002 ± 0.004 ^a^
		Ph-β-sitosterol with extract	5.19 ± 0.19 ^b^	14.9 ± 0.9 ^ef^	0.999 ± 0.004 ^a^
		Ph-ergosterol with extract	4.84 ± 0.54 ^b^	14.3 ± 0.5 ^f^	1.002 ± 0.004 ^a^

* The same letter in each column refers to the absence of statistically significant differences (for each variable separately) regarding the results of the statistical analysis in a one-way analysis of variance and Duncan’s post hoc test at *p* < 0.05 (n = 3). Liposomes with pure phospholipids, Ph; liposomes with phospholipids and 20 mol% of β-sitosterol or ergosterol, Ph-β-sitosterol and Ph-ergosterol, respectively.

**Table 4:** Diffusion coefficients (D) and diffusion resistance (R) of the fumitory extract and fumitory extract-loaded liposomes (non-treated and UV-irradiated samples) in simulated skin conditions (phosphate-buffered saline, pH 7.4, 35 °C). Liposomes with pure phospholipids, Ph-liposomes; liposomes with phospholipids and 20 mol% of β-sitosterol, Ph-β-sitosterol liposomes; liposomes with phospholipids and 20 mol% of ergosterol, Ph-ergosterol liposomes.

Samples		D (m^2^/s)	R (s/m)
Fumitory extract		5.09 × 10^−9^	8.01 × 10^5^
Non-treated	Ph-liposomes	3.48 × 10^−9^	1.17 × 10^6^
	Ph-β-sitosterol liposomes	4.02 × 10^−9^	9.48 × 10^5^
	Ph-ergosterol liposomes	4.30 × 10^−9^	1.43 × 10^6^
UV-irradiated	Ph-liposomes	5.42 × 10^−9^	7.51 × 10^5^
	Ph-β-sitosterol liposomes	1.10 × 10^−8^	4.23 × 10^5^
	Ph-ergosterol liposomes	9.64 × 10^−9^	3.69 × 10^5^

## Data Availability

The datasets generated during and/or analyzed during the current study are available from the corresponding author upon reasonable request.

## Supplementary Materials

The following supporting information can be downloaded at: https://www.mdpi.com/article/10.3390/pharmaceutics17060782/s1, Flow of calculations related to release kinetics title.
