# Supplementary material for: Nano-Liposomal Carrier as Promising Dermal Delivery Platform for Fumaria officinalis L. Bioactives
Source: Pharmaceutics. 2025 Jun 14;17(6):782. doi: 10.3390/pharmaceutics17060782 (PMC12196744; doi:10.3390/pharmaceutics17060782)
Supplement: Supplementary file 1 [file pharmaceutics-17-00782-s001.zip › pharmaceutics-3693191-supplementary.pdf]

Flow of calculations related to release kinetics

Results obtained from the release studies of fumitory extract and fumitory extract-loaded liposomes were analyzed to determine the diffusion coefficients (D) and diffusion resistances (R) derived from liposomes in simulated skin conditions (pH 7.4, 35ºC). The diffusion of polyphenols from liposomes to the receptor fluid through the membrane can be approximated using Fick's second law, shown in Equation (1):

$$\ln\left(\frac{C_d^0 - C_r^0}{C_d - C_r}\right) = D\,\beta\,t \quad (1)$$

where C_d and C_r are the concentrations of carob polyphenols detected in the donor and receptor compartments at time t; C_d^0 and C_r^0 are the concentrations of carob polyphenols at the beginning of the study; and D is the diffusion coefficient. The geometrical constant β value, typical for the Franz cell geometry, was 2.49×10^4 m^{-2}.

The diffusion coefficients of fumitory polyphenols from liposome dispersion or pure extract were calculated from the slope of the linear part of a curve defined by plotting $\ln\left(\frac{C_d^0 - C_r^0}{C_d - C_r}\right)$ *vs.* time.

The overall diffusion resistance, R, was calculated using Equation (2):

$$R = \frac{\delta}{D} \quad (2)$$

where δ is the membrane thickness.

Diffusion resistance represents the cumulative resistance of a semipermeable acetate cellulose membrane and the resistance of a liposomal bilayer. The contribution of the resistance, which is generated by the synthetic membrane, was determined from the diffusion of polyphenols from the pure fumitory extract. Then, the liposome resistance was determined by subtracting the synthetic membrane resistance from the overall diffusion resistance.
